# Effectiveness of a Transanal Drainage Tube for the Prevention of Anastomotic Leakage after Laparoscopic Low Anterior Resection for Rectal Cancer

**DOI:** 10.31557/APJCP.2020.21.5.1441

**Published:** 2020-05

**Authors:** Zheng Wang, Jianwei Liang, Jianan Chen, Shiwen Mei, Qian Liu

**Affiliations:** *Department of Colorectal Surgery, National Cancer Center/National Clinical Research Center for Cancer/Cancer Hospital, Chinese Academy of Medical Sciences and Peking Union Medical College, China. *

**Keywords:** Transanal drainage tube, anastomotic leakage, laparoscopic low anterior resection, rectal cancer

## Abstract

**Background and Objective:**

Anastomotic leakage is one of the most serious complications after laparoscopic low anterior resection Low Anterior Resection (LAR) for rectal cancers. The purpose of this study was to evaluate the effectiveness of a transanal drainage tube placed for the prevention of anastomotic leakage after laparoscopic LAR.

**Methods:**

The clinical data of 220 patients with rectal cancer who underwent laparoscopic LAR using the double stapling technique Double Stapling Department of Colorectal Surgery, National Cancer Center/National Clinical Research Center for Cancer/Cancer Hospital, Chinese Academy of Medical Sciences and Peking Union Medical College Technique (DST) from Jun 2017 to Dec 2018 were analyzed retrospectively at our institution. A transanal drainage tube was placed after anastomosis in 120 patients (TDT group). Another 100 patients were operated on without a transanal drainage tube (NTDT group). Clinicopathological and surgical factors, the frequencies of anastomotic leakage and re-operation after leakage were compared between the two groups.

**Results:**

Patient age, gender, body mass index, American Society of Anesthesiologists (ASA) score, previous abdominal surgery, intraoperative blood loss, tumor size, tumor stage, specimen length, distance of tumor from the anal verge, and operative time were comparable between the two groups. Overall rate of leakage was 4.5% (10/220). The frequency of leakage was 3.3% (4/120) in TDT group and was 6.0% (6/100) in NTDT group. The rate of leakage was significantly lower in TDT group (p**<**0.05). Furthermore, the re-operation rate for symptomatic anastomotic leakage was 50.0% (2/4) in TDT group, while in contrast it was 83.3% (5/6) in NTDT group. The rate of re-operation was lower in TDT group than NTDT group (p**<**0.05).

**Conclusions:**

The use of a transanal drainage tube in laparoscopic LAR for rectal cancer is a simple and effective method for prevention of anastomotic leakage and decreases the rate of re-operation after symptomatic leakage.

## Introduction

Recently, laparoscopic low anterior resection (LAR) with Total Mesorectal Excision (TME) has been commonly performed for middle and lower rectal cancers. With improvements in surgical techniques and perioperative management, the sphincter preservation rate has significantly increased without compromising oncological outcome, then the incidence of anastomotic leakage seems to increase at the same time (Peeters et al., 2005). Anastomotic leakage is one of the most serious complications following LAR for rectal cancers. Anastomotic leakage may cause serious morbidity, peritonitis, and sepsis. It may also affect patients’postoperative quality of life and lead to longer hospitalization, poor postoperative function, considerable extra cost and poor survival rates (Hallbook and Sjodahl, 1996; Nesbakken et al., 2001; Branagan et al., 2005; Law et al., 2007).

The frequency of symptomatic anastomotic leakage after LAR has been reported at 0.8–19.2 % (Milsom et al., 2009; Lam et al., 2011; Yamamoto et al., 2012; Kulu et al., 2013; Park et al., 2013). Some reports stated that the creation of a Diverting Stoma (DS) for proximal fecal diversion could reduce the incidence of symptomatic anastomotic leakage after LAR. Since DS blocked the passage of stool and gas through the anastomotic site, DS could reduce the endoluminal pressure in the anastomotic portion. It is suggested that the reduction of pressure in the anastomotic portion is very important for the prevention of anastomotic leakage and transanal drainage tube placement can reduce the pressure in the rectum. However, there are few studies and literature on the effectiveness of such transanal drainage tube placement. The purpose of this study was to evaluate the safety and effectiveness of a transanal drainage tube placed for the prevention of anastomotic leakage after laparoscopic LAR.

## Materials and Methods

*Patients*

Institutional review board approval was obtained before conducting this study. From Jun 2017 to Dec 2018, a total of 220 patients were diagnosed with rectal adenocarcinoma and underwent laparoscopic LAR using the double stapling technique in our hospital. All patients were operated on by the same colorectal surgical team. Patients who underwent a protective defunctioning stoma procedure, emergency operation or palliative operation, as well as patients who received prior chemoradiotherapy were excluded because they received DS. A transanal drainage tube was placed after anastomosis in 120 patients (TDT group). Another 100 patients were operated on without a transanal drainage tube (NTDT group). 

All the patients routinely underwent either pelvic Magnetic Resonance Imaging (MRI) or transrectal ultrasound and electronic colonoscope examination before surgery to identify the disease region and the pathologic type. All patients diagnosed with rectal adenocarcinomas after pathologic examination. The preoperative routine chest x-ray, abdominal ultrasound, and upper abdominal Computed Tomography (CT) examination showed no pulmonary, hepatic, or other distant metastases. 

The following aspects were recorded and investigated: patient age, gender, body mass index, ASA score, previous abdominal surgery, operation time, intraoperative blood loss, tumor size, tumor stage, specimen length, distance of tumor from the anal verge, and distance between the anastomosis line and the anal verge. The specimens were fixed unpinned, examined for margin clearance and staged according to the seventh edition of the American Joint Committee on Cancer (AJCC ) mamual. These parameters were compared between two groups. 

*Operative techniques*

All operations were performed by the same surgical team specializing in colorectal surgery. All the patients received bowel preparation. During the dissection of the rectum in laparoscopically assisted approaches, the principles regarding total mesorectal excision were followed including meticulous sharp dissection through the holy plane described by Heald et al and protecting the integrity of the fascial layer over the mesorectum (MacFarlane et al., 1993). The patient was put in the low Lloyd-Davies position, a 12-mm umbilical port was inserted and pneumoperitoneum established before 2 (12 and 5 mm) right-sided and 1 left-sided (5mm) ports were inserted. The small bowel was displaced from the pelvis with Trendelenderg and right tilt applied. The inferior mesenteric artery and vein were defined and transected with laparoscopic linear staplers. The lateral colonic attachments were then freed along the line of Toldt to completely mobilize the left colon. The sigmoid colon and rectum were mobilized down to the pelvic floor. The ureters, the hypogastric nerves, and the pelvic parasympathetic plexus were safeguarded. The rectum was transected at a level at least 50 mm distal to the inferior margin of the tumor in upper rectal cancers and 20 mm distal to the inferior margin of the tumor in middle and lower rectal cancers. An end to end anastomosis was performed using DST in all patients. We inserted an intra-abdominal drain around the anastomosis. The transanal drainage tube used in our hospital was a pleural drainage tube (36Fr). After anastomosis, an air leak test was performed in all patients. When the leak test was positive, the anastomotic site was repaired by suture until a negative result was obtained. The drainage tube was gently inserted into the anus, and positioned with the tip 30-50 mm proximal to the anastomotic site. The tube was fixed with a skin suture and connected to a drainage bag. In most cases, the tube was removed 7 days after the operation.

*Definition of anastomotic leakage*

Anastomotic leakage was defined as the presence of clinical symptoms such as fever or septicemia combined with the occurrence of pelvic abscess, presence of rectal pus discharge, formation of a rectovaginal fistula, or presence of peritonitis within 30 days after surgery, leading to a clinical and/or radiological examination (CT scan or enema examination using gastrografin) to confirm the leakage.

*Statistical analysis*

Statistical analysis were performed with SPSS software, version 15.0 for Windows (SPSS Inc., Chicago, IL, USA). Results were given as percentages, mean and standard deviations, or median and ranges. Quantitative and qualitative variables were compared with the Student t test and χ^2^ test, respectively. P-value of less than 0.05 was considered statistically significant. 

**Table 1:** Clinicopathological and Surgical Factors

	TDT group	NTDT group	*P*-value
	(n=120)	(n=100)	
Age (years)	58.6±11.3	57.3±10.1	0.086
Gender (male / female)	70/50	64/36	0.308
BMI(kg/m^2^)	25.8±6.1	24.7±5.3	0.084
ASA score			0.193
1	10 (8.3%)	8 (8.0%)	
2	72 (60.0%)	66 (66.0%)	
3	38 (31.7%)	26 (26%)	
Previous abdominal surgery	7	5	0.217
Operation time (min)	130.1±28.2	108.2±41.3	0.031
Intraoperative blood loss (ml)	20.5±10.9	20.3±12.4	0.42
Tumor size (cm)	4.1±1.5	3.8±1.2	0.794
Tumor stage			0.273
I	24	21	
II	90	76	
III	6	3	
Specimen length (cm)	25.6±5.3	24.5±4.9	0.832
Distance of tumor from the anal verge (cm)	6.8±2.0	9.1±2.9	**<**0.001
Distance between the anastomosis line and the anal verge (cm)	4.5 ± 1.7	5.5 ± 1.5	0.017

## Results

Between Jun 2017 to Dec 2018, a total of 220 patients with rectal cancer underwent laparoscopic LAR. There were 120 patients in TDT group and 100 patients in NTDT group. The clinicopathological and surgical data are shown in Table 1. For these data, such as patient age, gender, body mass index, ASA score, previous abdominal surgery, intraoperative blood loss, tumor size, tumor stage, specimen length, distance of tumor from the anal verge, and the distance between the anastomosis line and the anal verge, there were no significant differences between the two groups. The operation time was similar between the two groups. 

The anastomotic leakage of patients in the two groups was compared, anastomotic leakage occurred in four patients in TDT group and in six patients in NTDT group. The rate of anastomotic leakage was 3.3% in TDT group and 6.0% in NTDT group. TDT group had a significantly lower anastomotic leakage rate compared to NTDT group (p<0.05). The frequency of re-operation for anastomotic leakage was 50% in TDT group and 83.3 % in NTDT. The re-operation rate was significantly lower in TDT group than in NTDT group (p<0.05).

## Discussion

Many studies of the anatomy, pathology, biological characteristics, and lymph node metastasis mechanisms of rectal cancer, the introduction and popularization of the total mesorectal excision (TME) concept, specification of surgical procedures, and innovation of surgical instruments have all contributed to the positive oncologic and functional outcome for rectal cancer surgery. However, anastomotic complications after laparoscopic LAR have not been lessened with this surgical innovation. 

Anastomotic leakage is one of the most serious complications after LAR for rectal cancers and is considered a disaster to the colorectal surgeon. Anastomotic leakage commonly occurs in the early postoperative period, with a median time to leakage of 4–7 days (Kanellos, 2010). It has been reported that male gender, low tumor location, low anastomosis, preoperative chemoradiation, advanced cancer stage, longer operative time, blood loss, blood flow to the anastomosis, tension of the anastomosis, contamination of the operative field, the need for blood transfusions, and bowel preparation increased the risk of anastomotic leakage of rectal cancer surgery (Eberl et al., 2008; Lee et al., 2008; Bertelsen et al., 2010; Snijders et al., 2012; Park et al., 2013). Furthermore, endoluminal pressure in the anastomotic site is presumed to be associated with anastomotic leakage. In the early postoperative period, the anal sphincter is under a state of tight contraction and spasm from such factors such as pain, fear, inflammation, and trauma. This condition likely leads to a high endoluminal pressure, and the decompression of transanal drainage tube may therefore be in favour of the prevention of anastomotic leakage. There was no significant difference between the two groups with regards to the anastomotic leakage risk factors of gender and advanced cancer stage. However, the rate of anastomotic leakage was significantly lower in TDT group. In the light of this result, transanal drainage tube placement was very effective for prevention of anastomotic leakage after laparoscopic LAR. Gas and liquid stool in the rectum were effectively discharged through the transanal drainage tube. Consequently, the endoluminal pressure in the anastomotic site was reduced. This function of the transanal drainage tube may be a benefit in the prevention of anastomotic leakage. There are few studies and literature on the effectiveness of such transanal drainage tube placement. Our data are the first report concerning the safety and efficacy of the transanal drainage tube in laparoscopic LAR in China. In our study, the transanal drainage tube was made from an ordinary pleural drainage tube that had a relatively large diameter (36Fr). It was selected to provide adequate drainage, and positioned with the tip 30-50 mm proximal to the anastomotic site. The result of our study clearly shows that transanal drainage tube placement in laparoscopic LAR can reduce the rate of anastomotic leakage. 

In our institution, we routinely perform DS in laparoscopic LAR for patients with rectal cancer underwent preoperative radiotherapy for the prevention of anastomotic leakage. DS itself has clinical disadvantages such as patient discomfort, inconvenience, and the need for stoma closure surgery. If the efficacy of prevention of anastomotic leakage is nearly equal for both procedures, in the future we will use the method of transanal drainage tube placement for the prevention of anastomotic leakage in laparoscopic LAR for patients with rectal cancer underwent preoperative radiotherapy. 

Another procedure for the prevention of anastomoticleakage was transanal reinforcing suture after DST (Baek et al., 2013). The report stated that the rate of placement of DS was significantly lower in the reinforcing suture group compared with the non-suture control group, and no significant difference was observed in the rate of anastomotic leakage. The concept of transanal reinforcing suture was that the anastomotic site was reinforced by transanal suture, and might endure high pressure in the rectum. Transanal drainage tube placement added to reinforcing suture could further reduce the frequency of anastomotic leakage for super-low anterior resection.

The rate of re-operation for anastomotic leakage was 83.3 % in NTDT group and 50.0% in TDT group. Because the gas and liquid stool could be drained from the rectum through the transanal drainage tube, a slight amount of liquid stool might leak from the anastomotic fistula to the pelvic space resulting in localized peritonitis. The localized peritonitis was cured conservatively by the abdominal drainage tube. Several reports have stated that the transanal vacuum-associated drainage was an effective method for treating non-septic major anastomotic leakage following LAR (Glitsch et al., 2008; Sirois-Giguere et al., 2013; Srinivasamurthy et al., 2013). Shrinkage of the abscess cavity was brought about by a transanal vacuum-associated drainage tube inserted into the cavity.

Our results demonstrated that the use of a transanal drainage tube resulted in excellent outcomes with a low anastomotic leakage rate and a low re-operation rate for anastomotic leakage after laparoscopic LAR. We believe that the use of the transanal drainage tube in low anterior resection for rectal cancer may be a simple, safe, and effective method for preventing the occurrence of anastomotic leakage.
